# The Extended Postoperative Care-Score (EXPO-Score)—An Objective Tool for Early Identification of Indication for Extended Postoperative Care

**DOI:** 10.3390/jcm8101666

**Published:** 2019-10-12

**Authors:** Timo Iden, Amke Caliebe, Jochen Renner, Maj-Britt Hertz, Jan Höcker, Päivi Suvanto-Scholz, Markus Steinfath, Norbert Weiler, Matthias Gruenewald

**Affiliations:** 1 Department of Anesthesiology and Intensive Care Medicine, University Medical Centre Schleswig-Holstein, Campus Kiel, 24105 Kiel, Germany; 2 Institute of Medical Informatics and Statistics, Kiel University, University Medical Centre Schleswig-Holstein, Campus Kiel, 24105 Kiel, Germany; 3 Department of Anesthesiology and Intensive Care Medicine, Friedrich-Ebert-Hospital, 24536 Neumünster, Germany

**Keywords:** Intensive Care Unit, extended postoperative care, risk score, ICU Bed Shortage, objective allocation, patient pathway, patient safety

## Abstract

Extended postoperative care and intensive care unit capacity is limited and efficient patient allocation is mandatory. This study aims to develop an effective yet simple score to predict indication for extended postoperative care, as there is a lack of objective criteria for early prediction of admission to extended care in surgical patients. This prospective observational study was divided into two periods (Period 1: Extended Postoperative Care-Score (EXPO)-Score generation; Period 2: EXPO-Score validation) and it was performed at a tertiary university center in Germany. A total of 4042 (Period 1) and 2198 (Period 2) adult patients ≥ 18 years old receiving elective or emergency surgery were included in this study. After identifying patient- and surgery-related risk factors by an expert panel, the EXPO-Score was developed through logistic regression from data of Period 1 and validated in Period 2. Three risk factors are sufficient for generating a reliable predictive EXPO-Score: (1) the American Society of Anesthesiologists’ (ASA) physical status, (2) cardiopulmonary physical exercise status expressed in metabolic equivalents (MET), and (3) the type of surgery. The score threshold (0.23) has a sensitivity of 0.87, a specificity of 0.91, and an accuracy of 0.90 for predicting indication for extended postoperative care. The EXPO-Score provides a validated, early collectable, and easy-to-use tool for predicting indication of extended postoperative care in adult surgical patients.

## 1. Introduction

Appropriate patient allocation to the Intensive Care Unit (ICU) and similar units of extended postoperative care is important due to its high cost and limited capacity. Yet, preoperative allocation remains challenging due to the demanding volume of high-risk patients and lack of objective criteria. The European Society of Intensive Care Medicine (ESICM) recently listed patient selection for postoperative ICU therapy as the second most important unresolved issue in perioperative intensive care medicine, being only preceded by fluid therapy [1], and conducted a large survey on patient admission procedures in European ICUs [2].

With more than 300 million estimated patients undergoing surgery worldwide each year and increasing life expectancy, the number of patients with advanced age and multiple comorbidities is continuously growing [3]. Pearse et al. investigated a high-risk surgical population with an alarming conclusion: high-risk selection accounted only for 12.5% of surgeries, but 80% of deaths; less than 15% of these patients were ever transferred to an ICU, which suggests inadequate ICU resource provision [4]. Similarly, as compared to cardiac surgery with excessive postoperative ICU admission rate, only 15% to 35% of high-risk non-cardiac surgical patients are admitted to the ICU [4,5]. At the same time, the overall mortality rate of cardiac surgery patients is only 3.5% as compared to 12.2% in high-risk non-cardiac surgery [5]. While more standardized surgical and anesthesiological approaches in cardiac surgery may have contributed to its lower mortality rate, the alarming differences in ICU admission and mortality rates suggest inadequate ICU admission for non-cardiac surgery patients. In elective surgery, unplanned ICU admissions are associated with higher mortality rates in contrast to planned admissions (8% vs. 2%) [6]. Thus, there is an urgent need for improving preoperative prediction of postoperative extended care indication in order to plan and provide safe postoperative care within the limits of capacity and cost.

On the other hand, postoperative admission to high care units can be detrimental, as this possibly induces an increased risk for unnecessary invasive treatment, circadian rhythm disruption, and the development of delirium. Additionally, it might take away valuable therapy resources from other patients. Therefore, it is imperative to allocate postoperative patients to the most appropriate level of care [7,8,9]. Specific patient-related preconditions and surgery-related factors are known to be important in the allocation process [10].

Over the last couple of decades, there were many perioperative scoring systems described [10]. However, there is yet no scoring system available that combines patient- and surgery-related factors to preoperatively predict indication for extended postoperative care.

Therefore, the aim of this trial is to develop an objective, reliable, easy to implement scoring system to predict indication for extended postoperative care.

## 2. Experimental Section

### 2.1. Study Population

The study was performed at the University Medical Centre Schleswig-Holstein (UKSH), Campus Kiel, Germany. UKSH, as one of the largest medical centers in Europe, is a tertiary university hospital with all major surgical departments, including cardiac and transplant surgery. The study was registered on ClinicalTrials.gov (NCT 02663505) and approved by the responsible Ethics Committee (D 558/15; Nov/03/2015).

All of the patients ≥18 years (ASA status I-IV) receiving either elective or emergency surgery under general or regional anesthesia were included. Only the first procedure during the patient’s hospital stay was analyzed, all others were excluded to ensure data independence.

### 2.2. Primary Outcome: Indication for ICU

The primary outcome for this study was postoperative indication for ICU. Note that ICU indication is not equivalent to ICU admission and one might occur without the other, which was occasionally observed in our study, e.g., because of availability. Indication for ICU was evaluated prospectively (i.e., before admission to a postoperative care unit) by the responsible anesthesiologist via the indication catalogue (Table 1) directly after the end of the surgical procedure, while still in the operating room. For every patient, these objective, predefined criteria had to be checked. The aim of this procedure was to guarantee as much as possible that an objective decision about ICU indication was made, independent from the planning prior to the surgery or from the actual decision to which level of care each patient was admissioned. The anesthiologists were especially trained that an objective evaluation of the ICU indication was mandatory for this study.

### 2.3. Choice of Included Risk Factors

At first, an expert panel was formed with anesthesiologists and intensivists that were experienced in the complexity of preoperative assessment of postoperative level of care. Furthermore, one statistician for evaluation and generation of the study design was included in the research group. The panel attempted to identify potentially relevant perioperative patient- and surgery-related risk factors. General patient-related recorded data included age, sex, body mass index (BMI), ASA (‘American Society of Anesthesiologists’) physical status, preoperative hemoglobin value (if available), and physical activity in metabolic equivalents (MET). One MET is defined as the basal oxygen consumption rate of a 40-year-old 70 kg man and it equals 3.5 ml O_2_ per kg bodyweight per minute. MET are considered to be a convenient method for evaluating the individual functional capacity as machine-aided testing is not necessary [11]. As individual functional capacity is a well known predictor for intraoperative adverse cardiac events, it is meaningful to obtain this information from patients before surgery [12]. As perioperative adverse cardiac events are increased in patients that are unable to perform 4 MET of work in daily life, we chose 4 MET as the threshold [12]. Examples of such activity include climbing a flight of stairs or walking on level ground at four miles per hour [12]. This information was obtained from patients during routine premedication rounds. It has been previously shown that this method is safe and rather tends to under- than overestimate the actual individual functional capacity, resulting in a buffer of safety [13]. Table 2 lists clinical comorbidities recorded and deemed important for risk stratification.

The risk of surgery was determined by the type of surgery performed. All of the surgeries were classified into one of 12 categories: thoracic surgery with one-lung ventilation, upper abdominal surgery, hip/knee arthroplasty, large ear-nose-throat/maxillofacial tumor surgery, urogenital surgery, vascular surgery, (thoracic) endovascular aortic repair, cardiac surgery, non-cardiac surgery with planned ICU admission (e.g., liver transplantation), as well as miscellaneous minor (e.g., hernia surgery), intermediate (e.g., osteosynthesis), and major (e.g., exploratory laparotomy) surgeries. The three “miscellaneous” categories were introduced to limit categories to a reasonable number with comparable figures. Furthermore, the urgency of surgery was recorded. Emergency surgery was subdivided by targeted time till skin incision: N0 (immediately), N1 (≤2 h), N2 (≤6 h) and N3 (≤24 h).

Altogether, 16 risk factors for the prediction of ICU indication were considered: six general patient-related (sex, weight/height (BMI), age, ASA physical status, physical capacity fitness in MET, hemoglobin), eight serious comorbidities, surgical procedure, and urgency of surgery.

### 2.4. Study Design

Professional data sheets were designed while using the commercially available EvaSys evaluation and survey software and automatically imported with a scanner (EvaSys Survey and Evaluation Software, Electric Paper Evaluationssysteme GmbH, Lüneburg, Germany). The project and clinical background, as well as the data sheet, were then introduced to the entire physician staff of the Department of Anesthesiology and Intensive Care Medicine. The necessity of high compliance, completeness of acquired data, and objectiveness of ICU indication was stressed. The attending anesthesiologist completed the data sheet before (item “risk factors”) and at the end (item “indication for ICU”) of every surgical procedure.

The study included the following periods:

- Pilot period; staff training (data excluded).
- Extended Postoperative Care-Score (EXPO)-Score generation period (Period 1); data collection for 16 weeks.
- EXPO-Score validation period (Period 2); data collection for eight weeks.

### 2.5. Sample Size Rationale

In Period 1 of this study, we considered the above listed 16 variables for the prediction of ICU indication. An upper value of 50 events per candidate predictor is recommended as an adequate sample size for a reliable selection of predictors from a larger set of candidate predictors [14], corresponding to 16 × 50 = 800 patients with an indication for ICU in our study. A frequency of ICU admission of 200 patients per month was estimated from local retrospective clinical data, resulting in the predicted study duration of three to four months. For the validation Period 2 of the study, we chose a 2:1 ratio of the sample size for model selection and validation phase. as recommended [14].

### 2.6. Statistical Analysis

Statistical analyses were performed with the statistics software R, version 3.2.2 [15]. The groups “indication for ICU (yes/no)” were tested for differences in single variables by the Fisher´s exact or the Wilcoxon rank-sum test, as suitable. For categorical variables with more than two categories, the *p* value of the Fisher test was obtained by Monte-Carlo simulations with 1,000,000 simulations. Logistic regression was applied for modelling. Model selection was performed by backward selection and the BIC criterion with the function stepAIC of the package MASS [16].

The continuous influence variables hemoglobin and BMI were tested for nonlinear influence via spline regression with the package gam [17]. For both of the variables, either the non-linear term or the variable itself was not significant in all analyses, thus only linear dependencies were taken into account. All other variables were coded on a nominal scale. For factors with more than two levels, the function glht of the package multcomp was applied for posthoc analyses with the Tukey procedure [18].

Fivefold cross validation was used for model evaluation and internal validation. Diagnostic performance values were calculated with the package pROC which was also used to produce the ROC plots [19]. The Youden index was used as a threshold for the calculation of sensitivity, specificity, and accuracy. All performed tests were two-sided.

## 3. Results

The present study, including a total of 6342 analyzed patients, was performed between 2016 and 2017. The pilot period and Period 1 for score generation was initiated in February 2016. In the latter, a total number of 4497 patient datasets were collected. 374 had to be excluded due to missing inclusion criteria and 81 due to missing information on ICU indication. Therefore, data from 4042 patients were analyzed in study Period 1.

The validation Period 2 began in May 2017. A total number of 2421 datasets were recorded, of whom 202 due to exclusion criteria and 21 due to no ICU indication information were excluded, so that a total number of 2198 datasets were analyzed (see Supplementary Figure S1 for flow diagram).

In Period 1, 866 patients had an indication for ICU. Table 3 presents patient characteristics and results from univariate analysis for potential risk factors. Table 1 gives the ICU indications.

Multiple regression analysis was applied for EXPO-Score generation. It derived nine significant risk factors for ICU indication (Supplementary Table S1) in an internal validation procedure (fivefold cross validation). Table 4 shows the comparison between models with different numbers of included variables. It can be seen that the following three risk factors are sufficient for generating a reliable predictive EXPO-Score: (1) ASA physical status, (2) physical capacity in MET, and (3) type of conducted surgery. For practical application, only the values of these three risk factors have to be assessed for a patient and the model gives the probability that the patient will have need of postoperative ICU. This probability will in the following be termed EXPO-Score. The model including these three factors has a sensitivity of 0.87, a specificity of 0.91, and an accuracy of 0.90 for a threshold for probability of ICU indication of 0.23, which means that an EXPO-Score of 0.23 or higher predicts postoperative ICU indication. The AUC was 0.96 (0.95–0.96). For practical purposes, we created a table that shows the calculated EXPO-Scores for all possible combinations of ASA physical status, MET, and type of surgery, allowing for easily obtaining an EXPO-Score value for any given patient (Supplementary Table S2). Note that, in the surgery categories, two were included with a predefined and planned ICU admission. As these might bias the model, we performed an additional grey zone analysis, where the two categories were disregarded (Table 4). This analysis revealed very similar values as the EXPO-Score with a sensitivity of 0.82, specificity of 0.91, and an accuracy of 0.83.

The corresponding receiver operating characteristic (ROC) curve shows no relevant difference between three and more than three variables (Supplementary Figure S2). The predictive quality begins to decline slightly in the model using two variables. The parameters of the final model are given in Table 5, including corresponding odds ratios.

**Figure 1:**
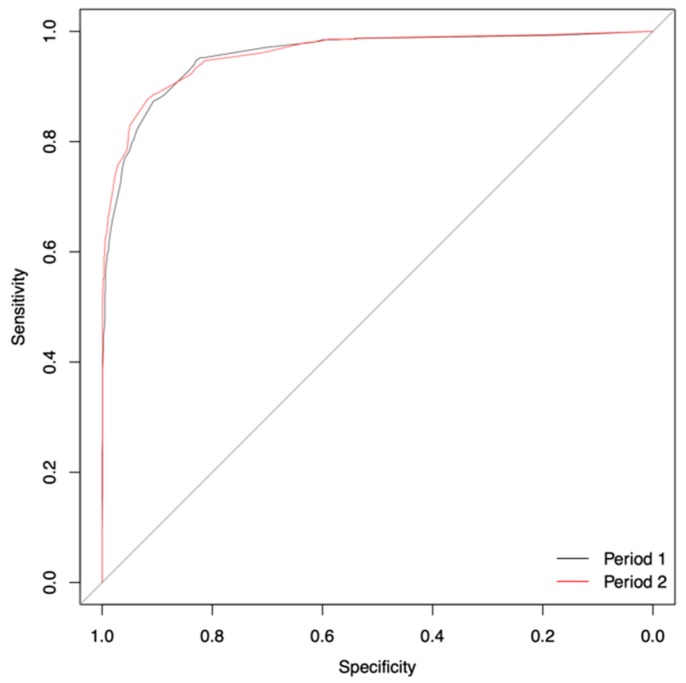
Receiver operating characteristic (ROC) curve Period 1 vs. Period 2.

The analysis of the validation Period 2 confirmed the previous results of the EXPO-Score with similar predictive performance and almost identical ROC curves (Figure 1 and Table 4).

## 4. Discussion

In this prospective study, we developed and validated the EXPO-Score, an objective, high precision, easily interpretable predictive tool for extended postoperative care indication in surgical patients. The EXPO-Score results in a continuous value between 0 (lowest risk) and 1 (highest risk). It only depends on three risk factors: ASA physical status, cardiopulmonary physical exercise status expressed in metabolic equivalents (MET), and the type of surgery. This makes the practical application of the EXPO-Score straightforward. The physician only has to collect information on these three easily obtainable variables. The EXPO-Score is easy to implement and can be obtained during routine premedication rounds without any special examinations, since no laboratory results or data from technical instruments are required. The underlying model is not complex and it can be integrated in a digital healthcare system or an app. For a patient in question the three risk factors have to be supplied to the software, and the output will be the EXPO-Score, i.e., the probability that this specific patient requires extended postoperative care.

Despite an existing consensus statement on the principles of ICU triage, evidence-based data on aligned allocation of ICU capacity for surgical patients is scarce and has not been widely explored [1,20]. Although available data suggest that inadequate or late ICU admission leads to higher mortality rates, yet clinicians often fail to identify patients with high perioperative risks and provide the appropriate postoperative level of care [5]. Conversely, over-admission to the ICU is costly [21] and can be detrimental, as ICU treatment is often stressful and invasive. Therefore, the early decision for adequate postoperative care logistics plays a crucial role in surgical patients.

The newly developed EXPO-Score predicts extended postoperative care indication with high accuracy. With a threshold of 0.23, i.e., a score of 0.23 or higher suggesting ICU indication, the scoring system optimizes both sensitivity and specificity. This means that the risk of allocating ICU care for a patient who will eventually not need it and the risk of allocating no ICU care for a patient who turns out to need it are equally weighted. Indeed, it is difficult to compare and evaluate the consequences of these two risks and this may depend on the patient population and on economic constraints. Therefore, we decided here to weigh them equally. Nonetheless, the threshold may be changed and adapted if that is desired by other health care systems with different external preconditions for extended postoperative care. A higher threshold would increase the risk of having no ICU care available for patients in need, but decrease the unnecessary assignment of ICU care. The key message of the score is not the calculated number itself, which might differ between hospitals and health care systems, but rather the opportunity to easily preoperatively design an objective priority list of high-risk surgical patients with the ultimate goal to properly plan extended high-care. Acute surgical complications are not always foreseeable but planning high-risk patients, as objectively identified by the score, at core operating room times when maximal personnel resources are available, can enhance patient safety. Moreover, scheduling patients with lower risks early in the morning can also optimize ICU bed use, i.e., if the reserved ICU bed remains unused by the first scheduled lower-risk patient, another high-risk surgery can still be started at core times. If done vice versa, surgery for the lower-risk patients cannot be started, as all postoperative high-care beds are already taken by the high-risk patients who had received surgery in the early morning.

The EXPO-Score is calculated from only three factors: ASA physical status, MET, and the type of surgery. The inclusion of other expert identified factors pertinent to extended care indication risk did not relevantly increase predictive performance.

The association between postoperative condition and patient ASA status and the type of surgery is well documented in the literature. The positive association between ASA physical status and postoperative mortality was originally published in 1970 [22] and recently confirmed in a large prospective study [23]. ASA status and the type of surgery were also identified to be associated with elevated postoperative cardiac risk in an observational study, including over 210,000 patients from the NSQIP database. Boersma et al. pointed out that using the type of surgery, instead of the simple distinction between high and non-high-risk surgery, yields superior cardiovascular mortality prediction [24]. Thus, it is not surprising that ASA physical status and the type of surgery are two of the factors included for EXPO-Score calculation [24,25]. MET, as an indicator of patient preoperative functional capacity, has been shown to predict perioperative risk, despite its self-reported nature [12,26]. A current study demonstrated that even a modest increase in physical activity expressed by MET was associated with a decrease in cardiometabolic risk [27]. These findings are consistent with our inclusion of MET into final EXPO-Score calculation. The hemoglobin value was a positive indicator for ICU admission in the univariate analysis, however inclusion did not generate additional statistical benefit (Supplementary Figure S3) and routine blood testing might not be recommended [28].

Of note, the EXPO-Score does not include opinions of patients or family members towards their desired level-of-care. In the ICE-CUB study including elderly patients, only 12.7% were asked for their opinion of the preferred level-of-care [29]. Heyland et al. demonstrated that patient requirements were not respected in 35% of the studied population, might lead to over-resuscitation [30]. It is evident that patient-centered care is important and we would like to stress including patient autonomy in the decision-making process.

Despite the conclusive results, a limitation of our study is its single-center design in a specific health care system. However, this study includes a high number of patients undergoing diverse surgical procedures. Further, we used well-defined, objective indicators for postoperative ICU in order to limit any personal preferences. Therefore, it is highly probable that the three most important risk factors identified and the scoring system produced are transferrable to other hospitals in health care systems equal or similar to ours. Adaptions might have to be made with regard to the probability threshold, above which extended or more intensive care is deemed necessary. Additionally, the extent and type of care may vary between hospitals or health care systems.

Additionally, we emphasize that the performed study was not based on retrospective data that were captured by electronic data acquisition, but prospective individual patient evaluation minimizing unreflective data. There were two categories with predetermined ICU admission (cardiac surgery and non-cardiac surgery with planned ICU admission) intentionally included in this study as a reflection of our current practice and to question this process. We additionally performed a grey zone analysis that excluded these two categories in order to investigate to what degree these patients influenced our results. This analysis showed the same specificity and comparable, slightly inferior values for sensitivity and accuracy (Table 4). Interestingly, we observed a few patients with planned ICU admission, who after operation had no indication for ICU. We conclude that the general message of this study is not blurred by including these patients with predetermined ICU admission.

The EXPO-Score assesses only surgical patients. Additional investigation needs to reveal whether it is of value for non-surgical patients.

In summary, there is growing interest in a preoperative objective tool for adequately allocating high-risk patients to extended postoperative care capacity. We have developed and validated such a reliable and easy-to-use scoring system: the EXPO-Score. We believe that it provides an evidence-based approach for predicting postoperative care indication and will assist clinicians in planning the appropriate level-of-care for surgical patients.

## Figures and Tables

**Table 1:** Indications for postoperative Intensive Care Unit (ICU) (primary outcome).

Indications for ICU Admission
Hemodynamic instability (e.g., vasopressor therapy) *n* = 372
Respiratory instability (e.g., re-intubation) *n* = 72
Massive intraoperative bleeding/transfusion *n* = 34
New severe cardiac arrhythmia (e.g., ventricular tachycardia) *n* = 15
Severe pre-existing disease (e.g., myasthenia, ejection fraction < 25%) *n* = 79
Surgery-related risk factor (e.g., liver transplantation) *n* = 479
Altered consciousness (e.g., delirium) *n* = 38
Hypothermia (body core temperature <36° Celsius) *n* = 13
High nursing care effort needed (e.g., immobility) *n* = 53
Other *n* = 108

*n* absolute frequencies observed in Period 1 (multiple indications possible).

**Table 2:** Clinical comorbidities deemed important for risk stratification.

Clinical Comorbidities
Cardiovascular (e.g., heart failure NYHA III-IV)
Pulmonary (e.g., COPD)
Liver cirrhosis/insufficiency (e.g., GOT/GPT > 2 × normal value)
Renal failure (e.g., creatinine > 200 µmol/L)
Neurologic disorders (e.g., stroke)
Sepsis/infection
Isolation/infection prevention (e.g., MRSA)
Endocrinopathy (e.g., complex diabetes mellitus)

*NYHA* New York Heart Association; *COPD* Chronic Obstructive Pulmonary Disease; *GOT* Glutamyl Oxaloacetic Transaminase; *GPT* Glutamyl Pyruvic Transaminase; *MRSA* Methicillin Resistant Staphylococcus Aureus; *ICU* Intensive Care Unit.

**Table 3:** Patient characteristics and risk factors.

Characteristics & Risk Factors	With Indication for ICU (*n* = 866)	No Indication for ICU (*n* = 3176)	*p* Value
**Sex**			<0.001
Men	556 (26.8)	1519 (73.2)	
Women	310 (15.8)	1657 (84.2)	
**Age** (years)			<0.001
<50	94 (7.4)	1179 (92.6)	
50–59	124 (18.3)	555 (81.7)	
60–69	210 (26.4)	584 (73.6)	
70–79	298 (31.9)	635 (68.1)	
80–89	124 (37.3)	208 (62.7)	
>90	16 (51.6)	15 (48.4)	
**BMI** kg/m^−2^ (Median (IQR))	26 (23–30)	26 (23–30)	0.49
**ASA**			<0.001
I	13 (1.9)	662 (98.1)	
II	90 (5.1)	1660 (94.9)	
III	592 (41.7)	828 (58.3)	
IV	171 (86.8)	26 (13.2)	
**Priority of Surgery**			<0.001
Elective	659 (19.2)	2769 (80.8)	
N0	51 (61.4)	32 (38.6)	
N1	53 (47.3)	59 (52.7)	
N2	63 (27.2)	169 (72.8)	
N3	33 (20.4)	129 (79.6)	
**Preconditions**			
Cardiovascular	514 (57.7)	377 (46.3)	<0.001
Pulmonary	124 (34.8)	232 (65.2)	<0.001
Liver insufficiency	24 (66.7)	12 (33.3)	<0.001
Renal insufficiency	71 (51.4)	67 (48.6)	<0.001
Endocrinopathy	64 (27.2)	171 (71.8)	0.033
Neurologic	112 (45.0)	137 (55.0)	<0.001
Infection/Sepsis	32 (69.6)	14 (30.4)	<0.001
Isolation/Infection (e.g., MRSA)	29 (70.7)	12 (29.3)	<0.001
Hemoglobin g/dl (Median [IQR])	13 (11–14)	14 (12–15)	<0.001
**Physical Activity**			<0.001
≥4 MET	486 (14.6)	2848 (83.4)	
<4 MET	376 (53.7)	324 (46.3)	
**Planned Postoperative Unit**			<0.001
Post anesthetic care unit	48 (1.6)	2983 (98.4)	
Intermediate care unit	27 (20.5)	105 (79.5)	
Intensive care unit	781 (91.8)	70 (8.2)	
**Planned surgery**			<0.001
Thoracic with OLV	13 (36.1)	23 (63.9)	
Upper abdomen	25 (37.9)	41 (62.1)	
Hip/knee arthroplasty	22 (15.7)	118 (84.3)	
Large ENT/maxillofacial tumor	23 (50.0)	23 (50.0)	
Urogenital	20 (16.9)	98 (83.1)	
Vascular	38 (35.2)	70 (64.8)	
Endovascular aortic repair	30 (75.0)	10 (25.0)	
Cardiac	300 (99.0)	3 (1.0)	
Non-cardiac with planned ICU	94 (94.9)	5 (5.1)	
Miscellaneous minor	94 (4.0)	2246 (96.0)	
Miscellaneous intermediate	132 (20.5)	513 (79.5)	
Miscellaneous major	73 (74.5)	25 (25.5)	

Patient characteristics and potential risk factors for ICU admission during study Period 1. Data are presented as *n* (% of “with” or “no” ICU indication within stratum) or otherwise indicated. The individual risk factor numbers do not necessarily add up to the total numbers because of occasional missing values. *p* values are the result of Fisher´s exact (categorical variables) or Wilcoxon rank-sum tests (continuous variables). *IQR* Interquartile range; *N0–N3* emergence categories (target time till skin incision): Immediately (N0), ≤ 2h (N1), ≤ 6h (N2) and ≤ 24h (N3); *MET* Metabolic equivalents; *OLV* One-lung ventilation; *ENT* ear-nose-throat.

**Table 4:** Model comparison.

Statistical Measure	Period 1								Period 2
	Number of Variables								
	9	7	6	5	4	3	2	Grey Zone	3
**Specificity**	0.91	0.90	0.91	0.87	0.89	0.91	0.88	0.91	0.92
**Sensitivity**	0.90	0.91	0.89	0.93	0.90	0.87	0.89	0.82	0.88
**AUC**	0.96	0.96	0.96	0.96	0.96	0.96	0.95	0.92	0.96
(95% CI)	(0.96–0.97)	(0.96–0.97)	(0.96–0.97)	(0.95–0.97)	(0.95–0.97)	(0.95–0.96)	(0.94–0.96)	(0.91–0.94)	(0.95–0.97)
**Accuracy**	0.91	0.90	0.91	0.88	0.89	0.90	0.88	0.83	0.91
**Threshold**	0.21	0.18	0.21	0.16	0.20	0.23	0.26	0.23	0.23

Comparison of models with different numbers of variables included. The columns with “3 variables” showing data for the selected Extended Postoperative Care-Score (EXPO)-Score model in Period 1 (Score generation) and Period 2 (Score validation). Grey zone analysis excludes surgical categories with planned ICU admission (“cardiac” and “non-cardiac with planned ICU”). *AUC* Area under the curve; *CI* Confidence interval.

**Table 5:** The final logistic model for the EXPO-Score.

Variable	Regression Coefficient	Standard Error	Odds Ratio	*p* Value
**ASA status**				
ASA I	Reference			
ASA II	0.40	0.37	1.49	0.27
ASA III	2.32	0.36	10.18	<0.001
ASA IV	4.56	0.43	95.58	<0.001
**Physical exercise status**				
≥4 MET	Reference			
<4 MET	1.26	0.15	3.53	<0.001
**Conducted surgery**				
Miscellaneous minor	Reference			
Miscellaneous intermediate	1.78	0.18	5.93	<0.001
Miscellaneous major	4.37	0.31	79.04	<0.001
Vascular	1.20	0.27	3.32	<0.001
Hip/knee arthroplasty	1.27	0.30	3.56	<0.001
Urogenital	1.61	0.31	5.00	<0.001
Thoracic with OLV	2.32	0.41	10.18	<0.001
Upper abdomen	2.95	0.33	19.11	<0.001
Endovascular aortic repair	3.51	0.43	33.45	<0.001
Large ENT/maxillofacial tumor	3.83	0.37	46.06	<0.001
Non-cardiac with planned ICU	6.79	0.50	888.91	<0.001
Cardiac	7.11	0.60	1224.15	<0.001

Final logistic model developed in Period 1 for the three selected variables (i) ASA status, (ii) physical exercise status and (iii) conducted surgery included in the EXPO-Score. The intercept for the regression model is −5.14 with a standard error of 0.35.

## Supplementary Materials

The following are available online at https://www.mdpi.com/2077-0383/8/10/1666/s1.
