# Supplementary material for: The Extended Postoperative Care-Score (EXPO-Score)—An Objective Tool for Early Identification of Indication for Extended Postoperative Care
Source: J Clin Med. 2019 Oct 12;8(10):1666. doi: 10.3390/jcm8101666 (PMC6832365; doi:10.3390/jcm8101666)
Supplement: Supplementary file 1 [file jcm-08-01666-s001.pdf]

Supplementary Materials: The following are available online at www.mdpi.com/xxx/s1

Supplementary Figure 1: Flow chart for study Period 1 and Period 2.

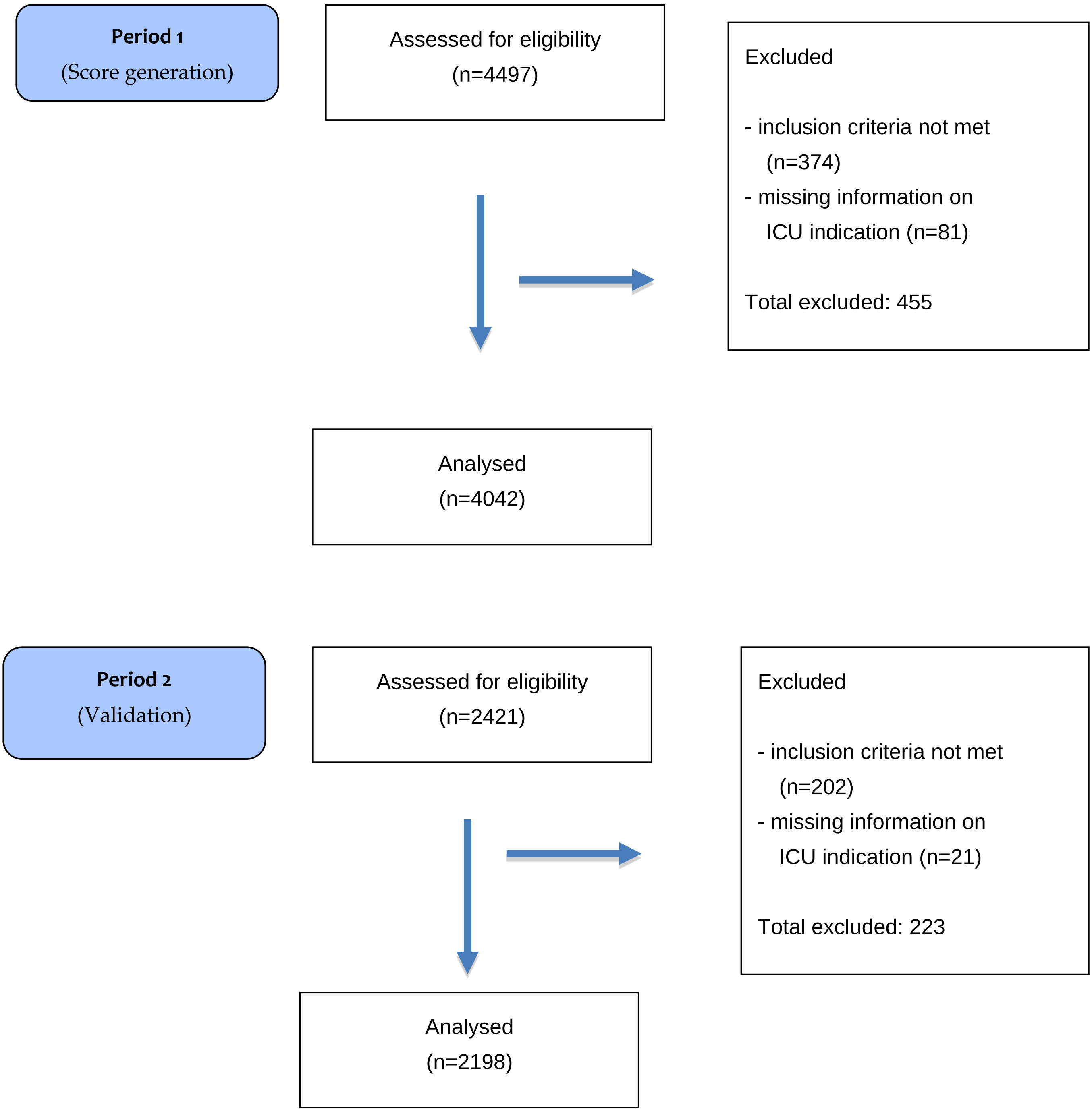

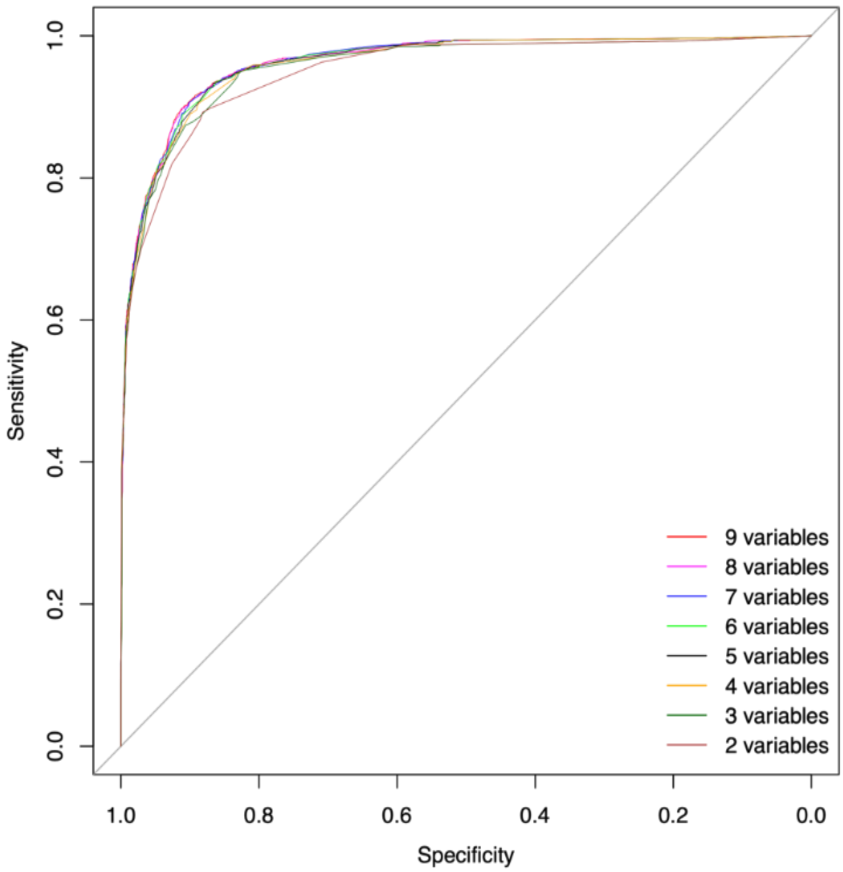

Supplementary Figure 2: Model comparison of 2-9 variables (ROC curve).

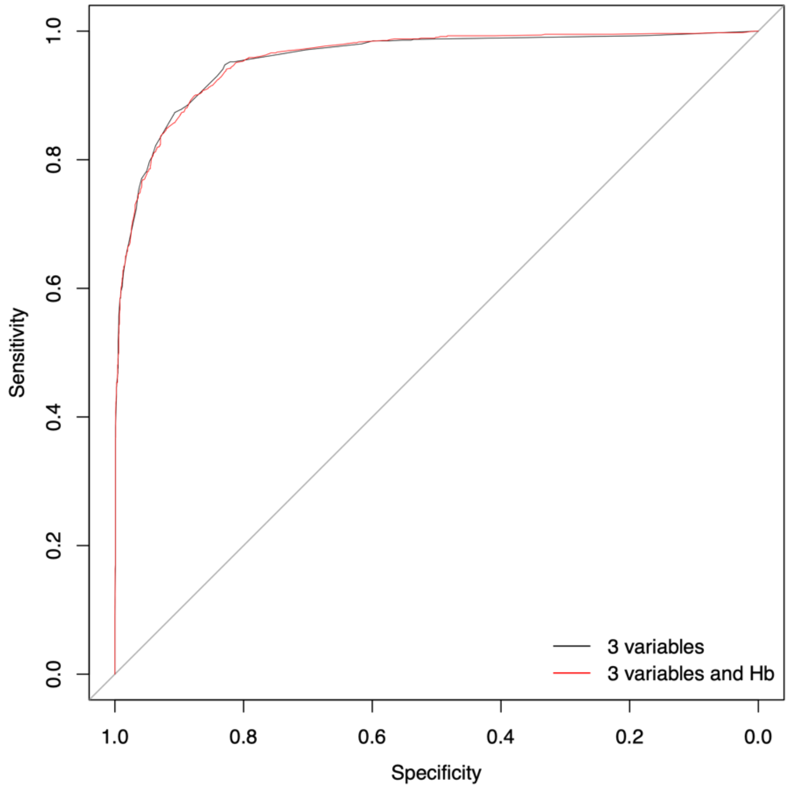

Supplementary Figure 3: Model comparison with and without Hb (ROC curve).

Supplementary Table 1: Results of the fivefold cross validation evaluation in Period 1.

Statistical Measure	Set 1	Set 2	Set 3	Set 4	Set 5	Ø	Total
**Threshold**	0.24	0.10	0.19	0.18	0.24	0.19	0.21
**Specificity**	0.92	0.84	0.90	0.90	0.89	0.89	0.91
**Sensitivity**	0.89	0.96	0.90	0.92	0.91	0.92	0.90
**AUC (95% CI)**	0.96 (0.94-0.97)	0.96 (0.94-0.97)	0.96 (0.94-0.97)	0.96 (0.95-0.98)	0.96 (0.95-0.98)	0.96	0.96 (0.96-0.97)
**Accuracy**	0.91	0.87	0.90	0.90	0.90	0.90	0.91
**Variables in model**	Urgency of surgery<br>ASA<br>Cardiovascular PC<br>Neurological PC<br>Isolation<br>MET<br>Kind of surgery<br>Infection<br>Sex	Urgency of surgery<br>ASA<br>Cardiovascular PC<br>Neurological PC<br>Isolation<br>MET<br>Kind of surgery<br>Infection<br>Liver insufficiency<br>Renal insufficiency	Urgency of surgery<br>ASA<br>Cardiovascular PC<br>Neurological PC<br>Isolation<br>MET<br>Kind of surgery<br>Infection<br>Sex	Urgency of surgery<br>ASA<br>Cardiovascular PC<br>Neurological PC<br>Isolation<br>MET<br>Kind of surgery<br>Sex	Urgency of surgery<br>ASA<br>Cardiovascular PC<br>Neurological PC<br>Isolation<br>MET<br>Kind of surgery<br>Sex<br>Liver insufficiency		Urgency of surgery<br>ASA<br>Cardiovascular PC<br>Neurological PC<br>Isolation<br>MET<br>Kind of surgery<br>Infection<br>Sex

Set i (for i between 1 and 5): predictive results for cross validation set i as test set for predictive performance; the other sets (train set) were used for model selection. Ø: average over the five sets. Total: results of model selection and predictive performance based on the complete dataset as both test and training set. The variable hemoglobin was not considered as influence variable in this analysis. *PC*—Precondition; *AUC*—Area under the curve; *CI*—Confidence interval; *MET* Metabolic equivalent

Supplementary Table 2: Calculated EXPO-Scores for all possible combinations of ASA physical status (ASA status I–IV), MET (1: ≥ 4 MET / 2: < 4 MET) and type of surgery (categories 1-12 as mentioned in paragraph 2.3). EXPO-Score shows values between 0 and 1; in order to receive percentages multiply score by 100.

ASA	MET	Surgery	EXPO-Score
**1**	1	1	0,211339482
**2**	1	1	0,28613144
**3**	1	1	0,730884988
**4**	1	1	0,962503542
**1**	2	1	0,485403538
**2**	2	1	0,585215292
**3**	2	1	0,905302799
**4**	2	1	0,989053829
**1**	1	2	0,020539377
**2**	1	2	0,030411953
**3**	1	2	0,175278341
**4**	1	2	0,667633985
**1**	2	2	0,068741076
**2**	2	2	0,099430557
**3**	2	2	0,427954181
**4**	2	2	0,876096265
**1**	1	3	0,056128195
**2**	1	3	0,081680546
**3**	1	3	0,376046129
**4**	1	3	0,850662851
**1**	2	3	0,173089977
**2**	2	3	0,238438103
**3**	2	3	0,679636789
**4**	2	3	0,952496233
**1**	1	4	0,100361605
**2**	1	4	0,143000334
**3**	1	4	0,530655073
**4**	1	4	0,9144286
**1**	2	4	0,281962962
**2**	2	4	0,370021915
**3**	2	4	0,799190403
**4**	2	4	0,974103659
**1**	1	5	0,028428387
**2**	1	5	0,041930607
**3**	1	5	0,228722397
**4**	1	5	0,737038919

**1**	2	5	0,093378913
**2**	2	5	0,133491234
**3**	2	5	0,510730458
**4**	2	5	0,907970475
**1**	1	6	0,019029493
**2**	1	6	0,028197213
**3**	1	6	0,16430148
**4**	1	6	0,65012964
**1**	2	6	0,063919045
**2**	2	6	0,092669954
**3**	2	6	0,409000902
**4**	2	6	0,867390075
**1**	1	7	0,163054782
**2**	1	7	0,225648092
**3**	1	7	0,663809016
**4**	1	7	0,94914046
**1**	2	7	0,406801283
**2**	2	7	0,506354158
**3**	2	7	0,874218364
**4**	2	7	0,985005398
**1**	1	8	0,838964226
**2**	1	8	0,886266949
**3**	1	8	0,981412964
**4**	1	8	0,998000193
**1**	2	8	0,948290006
**2**	2	8	0,964825705
**3**	2	8	0,994648416
**4**	2	8	0,999431062
**1**	1	9	0,87744385
**2**	1	9	0,914594188
**3**	1	9	0,986405888
**4**	1	9	0,998544
**1**	2	9	0,961834537
**2**	2	9	0,974157034
**3**	2	9	0,996100108
**4**	2	9	0,999585935
**1**	1	10	0,005805453
**2**	1	10	0,008658542
**3**	1	10	0,055874668
**4**	1	10	0,358708548
**1**	2	10	0,020140677

**2**	2	10	0,029827448
**3**	2	10	0,172404642
**4**	2	10	0,663179141
**1**	1	11	0,033593073
**2**	1	11	0,049423507
**3**	1	11	0,260518066
**4**	1	11	0,769039628
**1**	2	11	0,109019347
**2**	2	11	0,154703807
**3**	2	11	0,553590887
**4**	2	11	0,921388525
**1**	1	12	0,315395388
**2**	1	12	0,407963209
**3**	1	12	0,823606134
**4**	1	12	0,977841947
**1**	2	12	0,618563
**2**	2	12	0,708079729
**3**	2	12	0,942645599
**4**	2	12	0,993603675

Categories:

1) large ear-nose-throat/maxillofacial tumor surgery
2) hip/knee arthroplasty
3) thoracic surgery with one-lung ventilation
4) upper abdominal surgery
5) urogenital surgery
6) vascular surgery
7) endovascular aortic repair
8) non-cardiac surgery with planned ICU admission
9) cardiac surgery
10) miscellaneous minor
11) miscellaneous intermediate
12) miscellaneous major
